# Association Between Insulin Resistance and Remote Diffusion-Weighted Imaging Lesions in Primary Intracerebral Hemorrhage

**DOI:** 10.3389/fimmu.2021.719462

**Published:** 2021-07-29

**Authors:** Xiang-hua Ye, Jian-li Zhang, Yu-jia Jin, Dan Shen, Xiao-di Hao, Jia-wen Li, Jia-wei Zhong, Lu-hang Jin, Lu-sha Tong, Feng Gao

**Affiliations:** ^1^Department of Neurology, The Second Affiliated Hospital, Zhejiang University School of Medicine, Hangzhou, China; ^2^Department of Neurology, Lishui Hospital, Zhejiang University School of Medicine, Lishui, China; ^3^Department of Neurology, Henan Provincial People’s Hospital, People’s Hospital of Zhengzhou University, Zhengzhou, China

**Keywords:** intracerebral hemorrhage, insulin resistance, metabolism, remote diffusion-weighted imaging lesions, inflammation

## Abstract

**Background:**

Abnormal glucose metabolism was shown to be associated with the occurrence of remote diffusion-weighted imaging lesions (R-DWILs) after primary intracerebral hemorrhage (ICH) onset. Insulin resistance is a metabolic disorder that was regarded as an indicator of chronic systemic inflammation. In this study, we aimed to determine the effect of insulin resistance on the occurrence of R-DWILs in ICH.

**Methods:**

Patients with primary ICH within 14 days after onset were prospectively enrolled from November 2017 to October 2019. R-DWILs was defined as remote focal hyperintensity from the hematoma in DWI, with corresponding hypointensity in apparent diffusion coefficient. The homeostasis model assessment of insulin resistance (HOMA-IR) was used for insulin resistance estimation and calculated as fasting insulin (μU/ml) × fasting glucose (mmol/L)/22.5. Patients in our cohort were divided into four groups according to HOMA-IR index quartiles. Logistic regression analysis and smoothing plots were used to evaluate the association of HOMA-IR with R-DWIL occurrence. Sensitivity analysis was performed in non-diabetic patients, non-obese patients, hypertensive ICH patients, and patients 60 years and older separately. The association between HOMA-IR and systemic inflammatory immune indices neutrophil to lymphocyte ratio (NLR) and monocyte to lymphocyte ratio (MLR) was examined with multiple linear regression analysis.

**Results:**

Among the 345 patients, 54 (15.7%) had R-DWILs. Both the third and fourth quartiles of HOMA-IR index were robustly associated with an increased risk of R-DWIL occurrence (adjusted OR 3.58, 95% CI 1.33-9.65; adjusted OR 3.91, 95%CI 1.47-10.41) when compared with the first quartile. The association was consistent in non-diabetic, non-obese, hypertensive ICH patients, as well as in patients 60 years and older. Furthermore, both NLR and MLR were independently associated with HOMA-IR.

**Conclusions:**

Our study suggested that insulin resistance evaluated with HOMA-IR index was independently associated with the presence of R-DWILs in patients with acute and subacute primary ICH. It may provide new insights into the metabolism-related brain injury after ICH ictus.

## Introduction

Intracerebral hemorrhage (ICH) is one of the most prevalent subtypes of stroke and remains a significant cause of morbidity and mortality worldwide. Despite much efforts made in the past decades, no substantial improvement has emerged to improve functional outcomes after ICH (1). Nevertheless, glucose management has long been proposed to prevent unfavorable outcomes in both nondiabetic and diabetic patients with ICH, although no optimal criteria have been established (2). A better understanding of the impact of glucose metabolism on ICH is in urgent need. We have kept on focusing on the associations between glucose-related indices and the occurrence of remote diffusion-weighted imaging (DWI) lesions (R-DWILs) in brain MRI, which was characterized as hyperintensity lesions in DWI with corresponding hypointensity lesions in apparent diffusion coefficient (ADC), remote from hematoma location topographically (3, 4). Although commonly subclinical in the acute stage of ICH, R-DWILs portend worse outcomes as reported by previous studies (5–9). The etiology of R-DWILs is not fully elucidated. It is supposed to be a secondary injury of hemorrhage and probably related to cerebral local inflammation and blood brain barrier (BBB) disruption (3).

Our prior study discovered that increased admission fasting glucose, but not diabetes history, was positively associated with R-DWIL occurrence (3). It may be partially because of the prevalence of abnormal glucose metabolism in patients without diabetes but presenting with hyperglycemia under critical illness (4). Insulin resistance, a systemic metabolic disorder, and the hallmark of type 2 diabetes, is common in patients without diabetes but bearing vascular disease. Impaired insulin sensitivity has emerged as an important contributor to the development and unfavorable prognosis of ischemic stroke (10–15). The possible mechanisms may be attributed to hyperglycemia, hyperinsulinemia, dyslipidemia, hypertension, abnormal fibrinolysis, endothelia dysfunction, systemic inflammation, and atherogenesis (10). Moreover, insulin resistance is interconnected with neuroinflammation and is considered to be one of the major culprits in neurodegenerative process. Also, insulin resistance provides pro-inflammatory effects with profound consequences on the BBB (16–18). Therefore, we hypothesized that insulin resistance is associated with R-DWILs formation in ICH patients.

The homeostasis model assessment of insulin resistance (HOMA-IR) index is a well-established index and has been widely used to assess insulin resistance in clinical studies. In this study, we aim to clarify the hypothesis that insulin resistance measured by HOMA-IR index is associated with R-DWIL occurrence in acute and subacute stage of primary ICH.

## Materials and Methods

### Study Cohort and Participants

We prospectively enrolled patients with ICH within 14 days from onset, who were admitted to our hospital from November 2017 to October 2019. ICH was diagnosed based on the American Heart Association/American Stroke Association guideline (2) and confirmed by brain computed tomography (CT).

**Figure 1:**
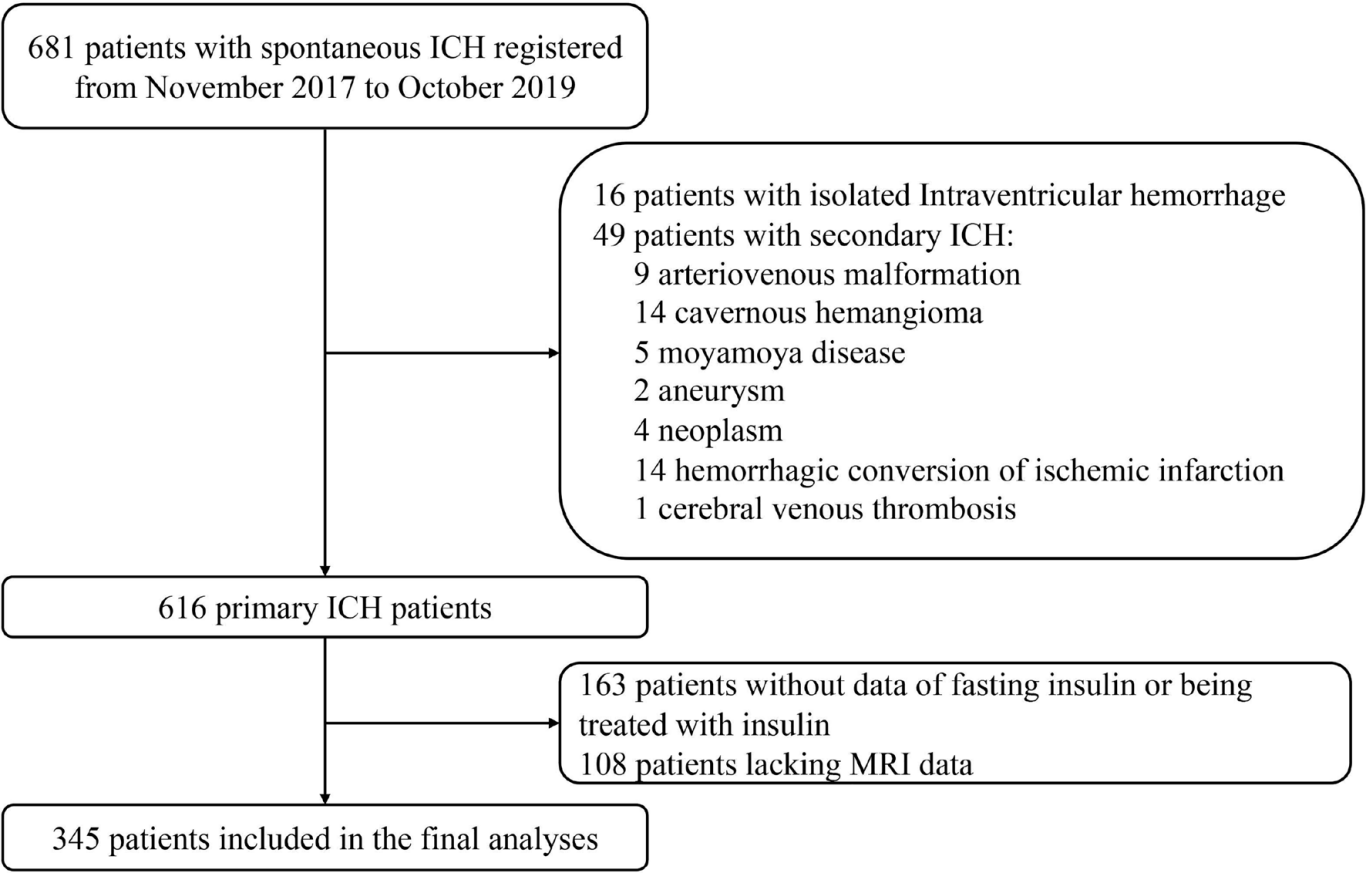
Flow chart of patient enrollment. ICH indicates intracerebral hemorrhage; MRI, magnetic resonance imaging.

Primary ICH patients were included in this study when brain MRI was operated within 28 days after onset with standardized protocol and reliable imaging data. Potential patients were excluded from the study if they presented solely intraventricular hemorrhage (IVH) or with secondary causes of ICH, such as arteriovenous malformation, cavernous hemangioma, Moyamoya disease, aneurysm, neoplasm, hemorrhagic conversion of ischemic infarction, or cerebral venous thrombosis. Patients with a recent head trauma were ineligible for our cohort. Those who were deficient in fasting insulin data or being treated with insulin before or during hospitalization were also ruled out (**Figure 1** in detail).

### Ethics Approval Statement

The work was approved by the institutional Human Research Ethics Committee of the Second Affiliated Hospital of Zhejiang University. Informed consents were waived due to the observational characteristic of this study, and the data are anonymous.

### Data Collection

Baseline demographic and clinical data, medical histories, vascular risk factors and medical therapies were recorded within 24 h after admission by trained neurologists. Demographic and clinical information included age, gender, height, weight, body mass index [BMI, calculated as the measured weight (kg) divided by the square of the measured height (m^2^)], time from symptom onset to hospitalization, time to take the MRI, and collect blood samples. Medical histories and vascular risk factors included history of hypertension, diabetes mellitus, atrial fibrillation (AF), ICH, cerebral infarction (CI)/transient ischemia attack (TIA), smoking, and drinking status. Medical treatments consisted of usage of antiplatelet drug, anticoagulant drug, antihypertensive drug, hypoglycemic agent, and statin before admission. The status of each patient’s neurological deficit was evaluated *via* the National Institutes of Health Stroke Scale score (NIHSS) by neurologists certified for assessing the scale.

The etiologies of ICH were categorized as hypertensive angiopathy (HA), cerebral amyloid angiopathy (CAA), and anticoagulation-related or undetermined cause. Two experienced neurologists (XHY and JLZ) determined the most likely etiology for the qualifying ICH based on available clinical data and neuroimaging, and reached consensus with a Kappa value of 0.98. The CAA-related ICH diagnosis was made according to the Boston criteria (19).

Blood samples were obtained in the next morning after admission with overnight fasting (at least 8 h), and routine laboratory examinations [absolute neutrophil count (ANC), absolute lymphocyte count (ALC), absolute monocyte count (AMC), total cholesterol (TC), low density lipoprotein cholesterol (LDL-C), creatinine, glucose, and insulin] were included. The estimated glomerular filtration rate (eGFR) was calculated as the following Chronic Kidney Disease Epidemiology Collaboration (CKD-EPI) equation for the Asian population: eGFR = 141 × min (serum creatinine/κ, 1) α × max (serum creatinine/κ, 1) −1.209 × 0.993 age × 1.018 [if female], where κ was 0.7 for females and 0.9 for males, α was −0.329 for females and −0.411 for males, min was the minimum of SCr/κ or 1, and max indicated the maximum of SCr/κ or 1 (20).

### Measurement of Insulin Resistance

Blood was collected at 2 days (IQR, 2–4 days) after ICH ictus. The blood levels of fasting glucose and insulin were measured using the hexokinase method and chemiluminescence immunoassay, respectively. Insulin resistance was estimated by the homeostasis model assessment of insulin resistance (HOMA-IR) with the following formula: fasting insulin (μU/ml) × fasting glucose (mmol/L)/22.5 (21). There was no clear definition of insulin resistance based on HOMA-IR index among different populations. The higher quartiles of HOMA-IR indicated more severe insulin resistance. Furtherly, a HOMA-IR score of more than 3.0 was used for diagnosing insulin resistance in non-diabetic patients in our study, as previously reported in IRIS Trial (22).

### Measurement of Systemic Inflammatory Immune Status

Neutrophil to lymphocyte ratio (NLR) and monocyte to lymphocyte ratio (MLR) are two widely used indicators for evaluating systemic inflammatory immune status. NLR was calculated as ANC divided by ALC. MLR was calculated as AMC divided by ALC (23).

### Neuroimaging Protocol

CT scan was performed on admission and in subsequent review if necessarily, using multidetector row scanners [Optima CT540, General Electric (GE) Healthcare, Connecticut, America; or SOMATOM Sensation 16, Siemens, German] with followed parameters: slice thickness, 5 mm; 120 Kv, and 100 to 300 mAs. MRI within 28 days after ICH onset was performed using 1.5-Telsa (T) (Sonata, Siemens, German) or 3.0-T scanner (Signa HDxt; GE Healthcare, Hartford, CT) with sequences comprised of axial T1-weighted, T2-weighted, T2 fluid attenuated inversion recovery (FLAIR), DWI [parameters on 1.5-T scanner: repetition time (TR) 3,100 ms, echo time (TE) 84 ms, b = 0/1,000 s/mm^2^, 6-mm slice thickness, 0.5-mm gap, field of view (FOV) 230×230 mm/3.0-T scanner: TR 5,200 ms, TE 75 ms, b = 0/1,000 s/mm^2^, 6-mm slice thickness, 0.5-mm gap, FOV 240 × 240 mm], apparent diffusion coefficient (ADC), and T2 star weighted angiography (SWAN).

### Assessment of Hematoma

The size and localization of hematoma were evaluated from initial head CT scan. Hematoma volume was calculated using the ABC/2 method (24). Hematoma location was classified into four types and described as lobar (including frontal, temporal, parietal, occipital, and insular lobe), deep (including basal ganglia and thalamus), infratentorial (brainstem or cerebellum), and mixed (two or more locations above involved). The ventricles and subarachnoid extension were also recorded if present. The collection of hematoma features was done by one certified neurologist (JWZ).

### Assessment of R-DWILs

**Figure 2:**
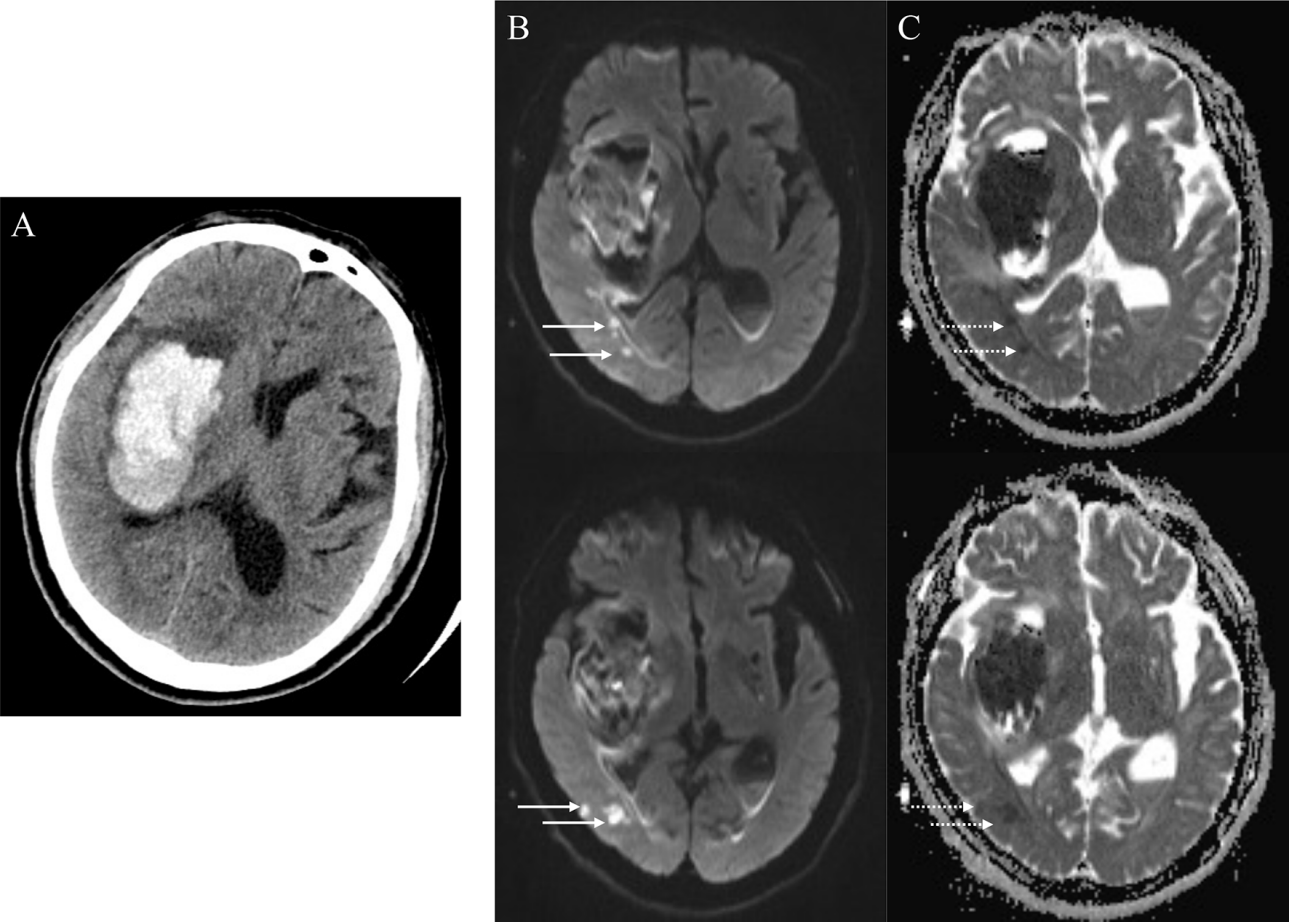
Positive remote diffusion-weighted imaging lesions in a 65-year old man with right basal ganglia hemorrhage **(A)**. Diffusion-weighted imaging shows hyperintensity lesions (**B**, solid arrowheads) in ipsilateral occipito-temporal lobe, with corresponding hypointensity in apparent diffusion coefficient map (**C**, dotted arrowheads) at the 8th day after ICH ictus.

As reported previously (3, 4, 25), R-DWILs was defined as a hyperintensity lesion in DWI, with corresponding hypointensity in ADC map, measuring less than 20mm in diameter (**Figure 2**). Restricted diffusion within or adjacent to the hematoma (< 20 mm) was not included in our study. DWI sequence and ADC map were read coherently to identify the R-DWILs. The data of R-DWILs was read independently by two experienced and trained readers (J.W.L and X.D.H) blinded to clinical and laboratory data. The inter-rater Cohen-weighted Kappa was 0.96 for the presence of R-DWILs.

### Assessment of Total Cerebral Small Vessel Diseases (cSVD) Burden

The assessment of cSVD burden was conducted as previously published (4, 26). Briefly, four MRI markers, including cerebral microbleeds (CMBs), white matter hyperintensities (WMHs), lacunes, and enlarged perivascular spaces (EPVS), was applied to measure cSVD severity. CMBs were defined as rounded or circular foci with low signal intensity on SWAN sequence with a diameter of 2 to 10 mm (27). WMHs were defined as signal abnormality in white matter with hypointensity in T1-weighted, and hyperintensity in T2-weighted and FLAIR sequences, rating visually on axial FLAIR images according to the four-point Fazekas scale (28). Periventricular white matter hyperintensity (PV-WMH) and deep white matter hyperintensity (D-WMH) were assessed, respectively; the overall degree of WMH was calculated as the total of PV-WMH and D-WMH scores. EPVS were defined as ≤2 mm round or linear CSF isointense lesions along the course of penetrating arteries. They were distinguished from lacunes by the size difference (lacunes >2 and ≤15 mm) and surrounding rim of FLAIR hyperintensity (29, 30). EPVS were counted on the brain slice showing the greatest extent of EPVS in the basal ganglia only. The evaluation of EPVS, lacunes, WMHs, and CMBs was performed by one experienced physician (XHY). Total cSVD burden was awarded as follows: one point was scored if there were presence of lacunes, >20 BG-EPVS, 1-4 CMBs, or three- to four- degrees WMHs, separately; two points was given if with ≥5 CMBs or five- to six-degree WMHs, respectively. The range of total cSVD score was therefore 0 to 6 (31).

### Statistical Analysis

Distributions of continuous variables were assessed for normality using the Shapiro-Wilk test. Continuous variables were expressed as median [interquartile range (IQR)] or mean [standard deviation (SD)] for non-normal or normal distributed variables, respectively. Categorical variables were presented as number (percentage). The index of HOMA-IR was classified into four subgroups according to the quartiles. The characteristics of patients were compared using ANOVA or the Kruskal-Wallis test for continuous variables and chi-squared test for categorical variables, among quartiles of HOMA-IR. When comparing the differences between patients with or without R-DWILs, *t* test, or Mann-Whitney *U* test was conducted for continuous variables and chi-squared test for categorical variables. Logistic regression modeling was used to evaluate whether HOMA-IR independently predicted the occurrence of R-DWILs after adjusting for potential confounders (age, gender, BMI, NIHSS, history of hypertension and diabetes mellitus, ventricle extension, total cSVD score, and antiplatelet use). Baseline variables, such as age, gender, BMI, history of hypertension, and diabetes mellitus, were assumed clinically relevant to the outcomes of interest, whereas other confounders were put into further analysis if their influence in effect were estimated more than 10%. The relationship between HOMA-IR and R-DWIL occurrence was also explored using smoothing plots. In the sensitivity analysis, we conducted analyses in non-diabetic, non-obese (BMI <25 kg/m^2^, a threshold recommended to identify obesity for Asians (32), hypertensive ICH patients, and patients 60 years and older, separately. The association of HOMA-IR with NLR and MLR was examined with Spearman analysis and multiple linear regression analysis.

All analyses were performed using the statistical package R (http://www.r-project.org) and EmpowerStats software (www.empowerstats.com, X&Y Solutions, Inc., Boston, MA). A two-tailed *p* value < 0.05 was considered to be statistically significant.

## Results

A total of 345 of 681 patients with ICH were finally enrolled in the study, after excluding those with isolated IVH, with secondary causes of hemorrhage, lacking insulin data or being treated with insulin, or with MRI unobtainable (**Figure 1**). Baseline characteristics of excluded patients because of missing insulin data or being treated with insulin are comparable with those who went into final analysis generally, except for the previous stroke/TIA occurrence, smoking status, and admission fasting glucose. When compared with patients excluded for lack of MRI data, the patients included in final analysis were less likely to experience severe neurological deficit, to have a history of AF, ICH, and smoking; had a higher BMI, TC, and LDL-C; and had smaller initial hematoma volume (**Supplementary Table S1**).

### Background Characteristics

Among the 345 patients for final analysis, the mean age was 61.1 ± 13.7 years, and 122 (35.4%) patients were women. A total of 99 lesions were found in 54 patients (15.7% in prevalence; range from 1 to 10 lesions per patient). The clinical and neuroimaging features of patients are reported in **Table 1**. The R-DWILs were more likely to occur in those who use antiplatelet drugs before ICH onset, those who presented ventricular hemorrhage, and those who had a higher HOMA-IR index or a higher total cSVD burden score. The median HOMA-IR was 2.11 (IQR, 1.33–4.04). Baseline characteristics of patients among different quartiles of HOMA-IR are summarized in **Supplementary Table S2**. Patients with higher HOMA-IR were younger, with a higher BMI and LDL-C level, and with higher score of NIHSS at admission, as well as being more likely to have a hypertension history and a higher initial blood pressure, with a higher frequency of diabetes, hypoglycemic treatments, and ventricle extension.

**Table 1:** Baseline characteristics of patients with and without R-DWIL.

	All patients	R-DWIL negative	R-DWIL positive	*p* Value
	(n=345)	(n=291)	(n=54)	
Age (years), mean (SD)	61.1 (13.7)	61.0 (13.9)	61.8 (12.9)	0.679
Female, n (%)	122 (35.4)	101 (34.7)	21 (38.9)	0.555
BMI (kg/m2), mean (SD)	24.0 (4.0)	24.0 (4.0)	24.1 (4.1)	0.622
NIHSS on admission, median (IQR)	4 (2, 10)	4 (2, 10)	4 (2, 10)	0.787
Systolic blood pressure (mmHg), mean (SD)	161.3 (25.5)	160.4 (24.6)	165.7 (30.0)	0.413
Diastolic blood pressure (mmHg), mean (SD)	91.0 (17.2)	90.4 (16.1)	94.2 (22.4)	0.601
History of hypertension, n (%)	265 (76.8)	219 (75.3)	46 (85.2)	0.112
History of diabetes mellitus, n (%)	65 (18.8)	52 (17.9)	13 (24.1)	0.284
History of atrial fibrillation, n (%)	10 (2.9)	7 (2.4)	3 (5.6)	0.409
History of ICH, n (%)	24 (7.0)	22 (7.6)	2 (3.7)	0.464
History of CI/TIA, n (%)	38 (11.0)	28 (9.6)	10 (18.5)	0.055
Smoking status, n (%)				0.556
Smoker or ex-smoker	101 (29.3)	87 (29.9)	14 (25.9)	
Non-smoker	244 (70.7)	204 (70.1)	40 (74.1)	
Drinking status, n (%)				0.322
Drinker or ex-drinker	116 (33.6)	101 (34.7)	15 (27.8)	
Non-drinker	229 (66.4)	190 (65.3)	39 (72.2)	
Medicine use before admission, n (%)				
Antiplatelet drug	31 (9.0)	19 (6.5)	12 (22.2)	0.001
Anticoagulant drug	7 (2.0)	6 (2.1)	1 (1.9)	0.92
Antihypertensive drug	155 (44.9)	126 (43.3)	29 (53.7)	0.158
Hypoglycemic drug	47 (13.6)	38 (13.1)	9 (16.7)	0.478
Statin	26 (7.5)	20 (6.9)	6(11.1)	0.422
TC (mmol/L), mean (SD)	4.8 (1.1)	4.7 (1.2)	4.9 (1.1)	0.473
LDL-C (mmol/L), mean (SD)	2.5 (0.8)	2.5 (0.8)	2.5 (0.7)	0.784
eGFR (ml/min/1.73m^2^), median (IQR)	99.9 (91.4, 109.8)	99.9 (91.1, 110.0)	100.8 (92.7, 109.5)	0.664
HOMA-IR, median (IQR)	2.1 (1.3, 4.0)	2.0 (1.3, 3.8)	3.0 (2.0, 4.6)	0.002
Hematoma volume (ml), median (IQR)	7.8 (3.0, 17.0)	7.8 (3.1, 16.6)	7.7 (2.7, 20.2)	0.789
Hematoma location, n (%)				0.361
Lobar	68 (19.7)	54 (18.6)	14 (25.9)	
Deep	214 (62.0)	181 (62.2)	33 (61.1)	
Infratentorial	44 (12.7)	41 (14.1)	3 (5.6)	
Mixed	19 (5.5)	15 (5.2)	4 (7.4)	
Ventricle extension, n (%)	102 (29.6)	78 (26.8)	24(44.4)	0.009
Subarachnoid extension, n (%)	31 (9.0)	27 (9.3)	4 (7.4)	0.855
Total cSVD burden, median (IQR)	2 (1, 3.8)	2 (1, 3)	3 (2, 4)	<0.001
Time to MRI (days), median (IQR)	6 (5, 8)	6 (5, 8)	6 (5, 8)	0.747
Presumed etiology of ICH, n (%)				0.268
HA	243 (70.4)	200 (68.7)	43 (79.6)	
CAA	40 (11.6)	36 (12.4)	4 (7.4)	
Anticoagulation or undetermined cause	62 (18.0)	55 (18.9)	7 (13.0)	

R-DWIL indicates remote diffusion-weighted imaging lesion; SD, standard deviation; BMI, body mass index; IQR, interquartile range; NIHSS, national institute of health stroke scale; ICH, intracerebral hemorrhage; CI, cerebral infarction; TIA, transient ischemic attack; TC, total cholesterol; LDL-C, low-density lipoprotein-cholesterol; eGFR, estimated glomerular fltration rate; HOMA-IR, homeostasis model assessment of insulin resistance; cSVD, cerebral small vessel disease; MRI, magnetic resonance imaging; HA, hypertensive angiopathy; CAA, cerebral amyloid angiopathy.

### Association Between HOMA-IR Index and Presence of R-DWILs

**Figure 3:**
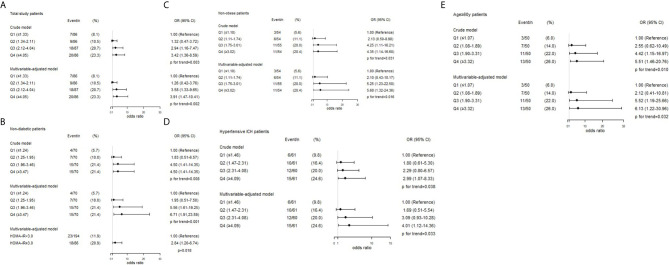
Associations between HOMA-IR index and the presence of R-DWILs in total study patients **(A)**, non-diabetic patients **(B)**, non-obese patients **(C)**, hypertensive ICH patients **(D)** and age ≥60y patients **(E)**. ORs and 95% CIs of R-DWILs occurrence are shown according to HOMA-IR quartiles in each group. The multivariable-adjusted models were adjusted for age (not for age ≥60y patients group), gender, body mass index (not for non-obese patients group), National Institutes of Health Stroke Scale score, history of hypertension, history of diabetes mellitus (not for non-diabetic patients group), ventricle extension, total cerebral small vessel disease score and antiplatelet use. OR indicates odds ratio; CI, confidence interval; Q, quartile; HOMA-IR, homeostasis model assessment of insulin resistance; R-DWILs, remote diffusion-weighted imaging lesions.

**Figure 4:**
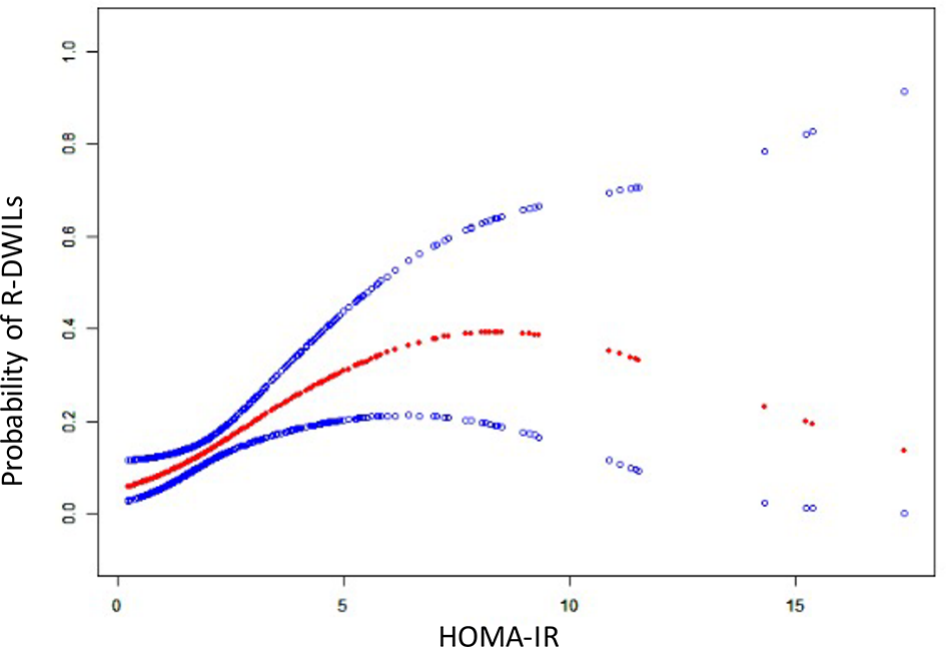
The relationship between HOMA-IR and the probability of R-DWILs. A nonlinear relationship between HOMA-IR and the probability of R-DWILs was observed after adjusted for age, gender, body mass index, National Institutes of Health Stroke Scale score, history of hypertension, history of diabetes mellitus, ventricle extension, total cerebral small vessel disease score and antiplatelet use. The probabilities and 95% CIs of R-DWILs occurrence are shown in red dotted line and blue dotted lines respectively.

The crude and adjusted association of HOMA-IR with R-DWILs are depicted in **Figure 3A**. The multivariable-adjusted ORs (95% CIs) of R-DWIL occurrence across the quartiles of HOMA-IR increased with a higher HOMA-IR range. Patients with a HOMA-IR index of Q3 and Q4 were associated with higher risk of R-DWIL occurrence when compared with patients with a HOMA-IR index of Q1. The smoothing plot was also used to observe the relationship between the HOMA-IR index and R-DWILs (**Figure 4**). We found that with the rise of HOMA-IR index, the risk of R-DWIL occurrence increased. The probability of R-DWILs reached a maximum when HOMA-IR closing to 10. However, as shown in the figure, the 95% CIs of R-DWIL occurrence for nine patients with HOMA-IR >10 were too large and the threshold effect was not reflected properly. We performed a sensitivity analysis, and the multivariable-adjusted ORs of R-DWIL occurrence showed a similar association in non-diabetic patients (**Figure 3B**), non-obese patients (**Figure 3C**), hypertensive ICH patients (**Figure 3D**), and patients 60 years and older (**Figure 3E**).

### Association Between Insulin Resistance and Presence of R-DWILs in Non-Diabetic Patients

Among the 280 non-diabetic patients, 86 (30.7%) had insulin resistance based on the HOMA-IR index cutoff of 3.0. Compared with patients without insulin resistance, those with insulin resistance were more likely to have R-DWILs (OR, 2.84; 95% CI, 1.20–6.74; *p*=0.018; **Figure 3B**) after adjustment with the potential confounders.

### Association of Insulin Resistance With NLR and MLR

After excluding patients with active infections before or during hospitalization, or with severe hepatic or renal diseases, 277 patients remained for analyses. NLR and MLR were both correlated with HOMA-IR. Multiple linear regression analysis was then applied and identified independent association of insulin resistance with NLR and MLR after adjusting for age, history of hypertension, statin use, and smoking (**Table 2**).

**Table 2:** Association of HOMA-IR index with NLR and MLR.

	HOMA-IR			
	Spearman analysis		Multiple linear regression analysis	
	r	*p* Value	Standardized coefficient	*p* Value
NLR	0.167	0.005	0.147	0.016
MLR	0.121	0.044	0.174	0.004

Spearman analysis and multiple linear regression analysis of HOMA-IR with NLR and MLR. Multiple linear regression analysis was adjusted for age, history of hypertension, statin use, and smoking. HOMA-IR indicates homeostasis model assessment of insulin resistance; NLR, neutrophil to lymphocyte ratio; MLR, monocyte to lymphocyte ratio.

## Discussion

In this study, we found that insulin resistance evaluated by HOMA-IR index was associated with R-DWIL occurrence in patients with primary ICH within 14 days after symptom onset. The association was consistent in patients without diabetes or obesity. Moreover, the significant relationship was also shown in patients with etiology of hypertensive angiopathy or in elderly patients.

Insulin resistance is a metabolic disorder resulting in wide-ranging effects on many organs and insulin-regulated pathways. It is characterized by a reduced action of insulin despite increased insulin concentrations (33). Emerging works indicated that insulin resistance prevents recovery from ischemic injury and subsequently worsened the functional outcomes after acute ischemic stroke (11–14). Insulin resistance also had adverse impact on non-diabetic patients with ischemic stroke or TIA (11, 12). However, existing evidence about the impacts of insulin resistance on ICH patients is scarce and controversial. In a large population-based Rotterdam study with 5,234 non-diabetic elderly enrolled, insulin resistance was not associated with the risk of ICH (34). The REasons for Geographic And Racial Differences in Stroke (REGARDS) study reported a nonsignificant yet discordant tendency of ICH risk in patients at higher levels of insulin resistance, with a downward trend in Whites and an upward trend in Blacks (35).

Our present work uncovered that insulin resistance was common not only in the overall ICH patients but also in non-diabetic and non-obese ICH patients. Because the HOMA-IR index is influenced by race, age, and baseline diseases, there is no universally accepted threshold to define insulin resistance with HOMA-IR. In this context, many investigators identified insulin resistance by the top quartile of HOMA-IR index in non-diabetic (11, 15, 34), others applied varied cutoffs (range, from 2.5 to 3.0) in discrepant populations among studies (22, 36). Interestingly, we found that patients in the fourth quartile of HOMA-IR along with a proportion of patients in the third quartile were indeed experiencing impaired insulin sensitivity, with reference of cutoffs mentioned above.

We previously confirmed that fasting blood glucose on admission was associated with the development of R-DWILs in ICH patients, whereas diabetes history showed no relation to it (3). It may partially be explained with the fact that glucose metabolic abnormality is common even among individuals without diagnosed diabetes (4). On the basis of this primary ICH cohort, we evaluated the association of insulin resistance with the presence of R-DWILs and reached a statistical correlation. Because diabetes and obesity are two metabolic syndrome components that closely correlated with insulin resistance, we also conducted analysis among non-diabetic and non-obese patients and yielded out similar results.

It is recognized that chronic low-grade inflammation is involved in the pathogenesis of insulin resistance, and in turn insulin resistance can reflect existing systemic inflammatory status (37). In our study, HOMA-IR was positively associated with systemic inflammatory immune indices NLR and MLR, meaning patients with severe insulin resistance suffering increased systemic inflammation. In the setting of ICH, already destroyed BBB allows invasion of peripheral immune cells and proinflammatory cytokines to the brain. Increased neuroinflammation results in further disruption of BBB (16, 17). Patients with insulin resistance were supposed to suffer from more significant BBB integrity deterioration. Moreover, dysregulated insulin signaling in brain activates NFκB transcription factors aggravating neuroinflammation (18). Inflammatory injury with abnormal tissue environment and remote extension of hematoma constituents through perivascular and perineural space were speculated as potential mechanisms of R-DWILs (3, 25).

The endothelial dysfunction also contributes to R-DWILs formation in ICH patients with insulin resistance. Because of the insulin receptor expressed in endothelium, insulin acts on vasculature as well, in addition to classical insulin target tissues like liver, skeletal muscle, and white adipose tissue (33). Under normal conditions, insulin acts on the insulin receptor of endothelial cells, leading downstream to phosphatidylinositol 3-kinase (PI3K)-Akt activation. Akt phosphorylates endothelial nitric oxide (NO) synthase to catalyze the production of NO from l-arginine, thereby resulting in vessel relaxation (38). However, under insulin resistance status, this pathway is impaired, whereas the mitogen-activated protein kinase (MAPK) pathway is preponderant and vasoconstriction is stimulated. What is worse, the activated endothelia express adhesion proteins, such as vascular cell adhesion molecule-1 and E-selectin, attract leukocytes and activate platelets, altogether leading to elevated permeability of the endothelia and potentially the development of local atherosclerosis (33). R-DWILs may be *in situ* small vessel occlusion implying an ischemia-prone state after ICH and may be partially due to this insulin resistance-related vessel injury (25).

Adequate cerebral perfusion is essential for brain function, whereas improper blood supply could lead to ischemia and brain damage within minutes. Cerebral autoregulation is one of the well-developed mechanisms in the brain to prevent from fluctuating of cerebral blood flow (39). Recent study has suggested that in acute ICH patients, with elevating intracranial pressure, the dynamic cerebral autoregulation was globally impaired within about 2 weeks (40). Among several elements contributing to the cerebral autoregulation, myogenic response, the ability of smooth muscle cells reacting to blood pressure changes, is the most prominent one (39). Studies in rats demonstrated that insulin resistance provoked an increase in cerebrovascular myogenic tone and a decrease in lumen diameter (41, 42), which could partly explain the increased risk of R-DWILs in patients with more severe insulin resistance after ICH. Besides, distal arteries possess a substantially higher myogenic activity than proximal arteries (39) and could be more vulnerable to insulin resistance-induced damage. Consistent with it, R-DWILs are mainly located in cortical or subcortical regions and are mostly small, dot-like.

HA and CAA are the two main etiologies for primary ICH. A few studies showed that R-DWILs were more commonly seen in patients with CAA-related ICH than in patients with HA-related ICH (43, 44). Although in other studies, similar occurrence of R-DWILs were observed in these two etiologies of ICH (45). Insulin resistance is a systemic metabolic abnormality, which has been proven to be related to other metabolic disorders closely, including hypertension (36). Insulin resistance is also associated with an increased level of amyloid-β 42 (Aβ42) in Alzheimer disease (AD) brains (16). Meanwhile, Aβ deposition in arteries is regarded as the cause of CAA. Because insulin resistance is correlated with both hypertension and accumulation of Aβ, we wonder if ICH patients with different etiologies may possess varied insulin resistance severity. We tried to explore the associations of R-DWIL occurrence with insulin resistance under different etiologies of ICH but yielded out null results.

In recent studies, piles of evidence revealed that the occurrence of R-DWILs was correlated with the total burden of cSVD. In our former study, we found that R-DWILs were more likely to present in patients with higher total cSVD burden (26). In a previous study in elderly, a nondiabetic, healthy population showed that insulin resistance was a major risk factor of cSVD burden (46). Thus, it is necessary to identify if cSVD burden would play a role in the association between insulin resistance and R-DWILs. Our present work found a meaningful association between cSVD burden and R-DWIL occurrence. However, we did not find a statistical relationship between HOMA-IR and cSVD burden, which may be partially because of the difference in study cohort and cSVD burden scoring methodology. Moreover, after being adjusted with potential confounders, including total cSVD score, the independent association between insulin resistance and R-DWILs still existed. It gave a reason to assume that insulin resistance was correlated with R-DWIL occurrence, independent of cSVD burden.

Besides, the ventricle extension of hemorrhage may play a role in the association between insulin resistance and R-DWILs. Elevated intraventricular concentrations of inflammatory markers were observed in ICH patients accompanied with IVH (47). Blood constitutes, inflammatory cells, and proinflammatory factors could extend through cerebrospinal fluid, resulting in brain injury in remote areas. In patients with severe insulin resistance in our cohort, a higher frequency of IVH was detected. The underlying mechanism is unclear, and further explorations are needed. However, the global neuroinflammation and deteriorated BBB in the insulin resistance setting was supposed to be coordinated with IVH regarding to R-DWIL occurrence.

This study has several limitations. Because of missing insulin data or being treated with insulin or lacking MRI data, 271 patients were ruled out from our study. The baseline characteristics between the patients included in and excluded from the analysis were not well balanced, especially those excluded for unavailable MRI data were more likely to suffer from fatal hemorrhage and experience severe clinical symptoms. This may lead to selection bias, whereas generalizability of the findings should be further validated in cohort, including severe cases. Another possible criticism of our study is that we collected blood sample for insulin sensitivity measurement too soon after ICH onset, which may not represent actual steady-state values. The cerebrovascular event itself may temporarily reduce insulin sensitivity, and then the insulin resistance prevalence may be overestimated (48). Although HOMA-IR correlated strongly with insulin resistance estimated by the gold standard of euglycemic-hyperinsulinemic clamp and is the most widely accepted surrogate measure of insulin resistance, there should be a cautious interpretation of the current finding.

## Conclusions

Our study demonstrated that insulin resistance was associated with the occurrence of R-DWILs in ICH patients within 14 days after symptom onset. This result shed light on insulin resistance as a potential metabolic factor involved in brain injury after ICH.

## Data Availability Statement

The raw data supporting the conclusions of this article will be made available by the authors, without undue reservation.

## Ethics Statement

The studies involving human participants were reviewed and approved by the institutional Human Research Ethics Committee of the Second Affiliated Hospital of Zhejiang University. Written informed consent for participation was not required for this study in accordance with the national legislation and the institutional requirements.

## Author Contributions

X-HY brought up the main idea, developed the protocol, collected and analyzed data, and wrote the manuscript. J-LZ collected the data, searched for literatures, and provided helpful input on the theme. Y-JJ helped analyzing data, found some useful papers, and offered some helpful suggestions. DS collected part of the data and searched for useful papers. J-WL, X-DH, and J-WZ helped to read the images and collected part of the data. L-HJ collected part of the data. FG and L-ST helped developing the protocol, supervised and offered guidance to all the authors, revised the manuscript, and polished the language. All authors contributed to the article and approved the submitted version.

## Funding

This work was supported by grants from the National Natural Science Foundation of China (NSFC) (81971155) to L-ST, the National Natural Science Foundation of China (NSFC) (81471168) to FG, and Science and Technology Action Plan for Major Diseases Prevention and Control in China to FG (2017ZX-01S-006S3).

## Conflict of Interest

The authors declare that the research was conducted in the absence of any commercial or financial relationships that could be construed as a potential conflict of interest.

## Publisher’s Note

All claims expressed in this article are solely those of the authors and do not necessarily represent those of their affiliated organizations, or those of the publisher, the editors and the reviewers. Any product that may be evaluated in this article, or claim that may be made by its manufacturer, is not guaranteed or endorsed by the publisher.
