# Supplementary material for: Association Between Insulin Resistance and Remote Diffusion-Weighted Imaging Lesions in Primary Intracerebral Hemorrhage
Source: Front Immunol. 2021 Jul 29;12:719462. doi: 10.3389/fimmu.2021.719462 (PMC8358397; doi:10.3389/fimmu.2021.719462)
Supplement: Supplementary file 1 [file Table_1.docx]

**Table S1.** Baseline characteristics of individuals included and excluded

| Characteristics | patients for full analysis  (n=345) | Excluded for missing insulin or being treated with insulin  (n=163) | *p* Value ***** | Excluded for missing MRI  (n=108) | *p* Value **ƚ** |
| --- | --- | --- | --- | --- | --- |
| Age (years), mean (SD) | 61.1 (13.7) | 62.3 (12.6) | 0.360 | 63.2 (14.9) | 0.179 |
| Female, n (%) | 122 (35.4) | 50 (30.7) | 0.297 | 39 (36.1) | 0.887 |
| BMI (kg/m2), mean (SD) | 24.0 (4.0) | 24.0 (3.4) | 0.765 | 23.0 (4.1) | 0.027 |
| NIHSS on admission, median (IQR) | 4 (2, 10) | 4 (2, 11) | 0.997 | 8 (3,15) | <0.001 |
| History of hypertension, n (%) | 265 (76.8) | 118 (72.4) | 0.280 | 74 (68.5) | 0.083 |
| History of diabetes mellitus, n (%) | 65 (18.8) | 25 (15.3) | 0.334 | 13 (12.0) | 0.102 |
| History of atrial fibrillation, n (%) | 10 (2.9) | 6 (3.7) | 0.637 | 10 (9.3) | 0.011 |
| History of ICH, n (%) | 24 (7.0) | 15 (9.2) | 0.364 | 15 (13.9) | 0.021 |
| History of CI/TIA, n (%) | 38 (11.0) | 9 (5.5) | 0.048 | 10 (9.3) | 0.644 |
| Smoking status, n (%) |  |  | 0.001 |  | 0.006 |
| Smoker or ex-smoker | 101 (29.3) | 72 (44.2) |  | 47 (43.5) |  |
| Non-smoker | 244 (70.7) | 91 (55.8) |  | 61 (56.5) |  |
| Drinking status, n (%) |  |  | 0.268 |  | 0.177 |
| Drinker or ex-drinker | 116 (33.6) | 63 (38.7) |  | 44 (40.7) |  |
| Non-drinker | 229 (66.4) | 100 (61.3) |  | 64 (59.3) |  |
| TC (mmol/L), mean (SD) | 4.8 (1.1) | 4.5 (1.2) | 0.064 | 4.5 (1.2) | 0.034 |
| LDL-C (mmol/L), mean (SD) | 2.5 (0.8) | 2.4 (0.9) | 0.074 | 2.3 (0.8) | 0.015 |
| FG (mmol/L), median (IQR) | 5.9 (5.2, 7.2) | 5.7 (4.9, 6.6) | 0.022 | 6.2 (5.3, 7.4) | 0.226 |
| Hematoma volume (ml), median (IQR) | 7.8 (3.0, 17.0) | 8.5 (3.0, 17.5) | 0.891 | 11.7 (5.3, 27.3) | 0.001 |
| Systolic blood pressure (mmHg), mean (SD) | 161.3 (25.5) | 159.2 (26.6) | 0.295 | 161.3 (31.4) | 0.879 |
| Diastolic blood pressure (mmHg), mean (SD) | 91.0 (17.2) | 90.0 (16.8) | 0.727 | 88.3 (19.7) | 0.089 |

MRI indicates magnetic resonance imaging; SD, standard deviation; BMI, body mass index; IQR, interquartile range; NIHSS, national institute of health stroke scale; ICH, intracerebral hemorrhage; CI, cerebral infarction; TIA, transient ischemic attack; TC, total cholesterol; LDL-C, low-density lipoprotein-cholesterol; FG, fasting glucose.

***** *p* value of comparison between included patients and patients excluded for missing insulin or being treated with insulin.

**ƚ** *p* value of comparison between included patients and patients excluded for missing MRI.
