# Supplementary material for: Association Between Insulin Resistance and Remote Diffusion-Weighted Imaging Lesions in Primary Intracerebral Hemorrhage
Source: Front Immunol. 2021 Jul 29;12:719462. doi: 10.3389/fimmu.2021.719462 (PMC8358397; doi:10.3389/fimmu.2021.719462)
Supplement: Supplementary file 2 [file Table_2.docx]

**Table S2.** Baseline characteristics of patients according to HOMA-IR index

|  | HOMA-IR | | | |  |
| --- | --- | --- | --- | --- | --- |
|  | Q1 (≤1.33) | Q2 (1.34-2.11) | Q3 (2.12-4.04) | Q4 (≥4.05) | *p* Value |
|  | (n=86) | (n=86) | (n=87) | (n=86) |  |
| Age (years), mean (SD) | 65.1 (12.9) | 60.8 (13.5) | 60.2 (13.7) | 58.5 (14.0) | 0.011 |
| Female, n (%) | 22 (25.6) | 31 (36.0) | 39 (44.8) | 30 (34.9) | 0.071 |
| BMI (kg/m2), mean (SD) | 21.8 (3.3) | 23.6 (3.1) | 24.0 (3.6) | 26.5 (4.5) | <0.001 |
| NIHSS on admission, median (IQR) | 3 (1, 9) | 4 (2, 8) | 6 (2, 11) | 5 (2, 12) | 0.032 |
| Systolic blood pressure (mmHg), mean (SD) | 155.4 (24.0) | 162.5 (25.2) | 165.3 (28.6) | 161.7 (23.4) | 0.047 |
| Diastolic blood pressure (mmHg), mean (SD) | 86.5 (14.8) | 91.7 (16.1) | 91.8 (20.0) | 93.9 (17.0) | 0.009 |
| History of hypertension, n (%) | 56 (65.1) | 66 (76.7) | 72 (82.8) | 71 (82.6) | 0.019 |
| History of diabetes mellitus, n (%) | 6 (7.0) | 9 (10.5) | 17 (19.5) | 33 (38.4) | <0.001 |
| History of atrial fibrillation, n (%) | 0 (0) | 1 (1.2) | 5 (5.7) | 4 (4.7) | 0.074 |
| History of ICH, n (%) | 8 (9.3) | 5 (5.8) | 6 (6.9) | 5 (5.8) | 0.782 |
| History of CI/TIA, n (%) | 7 (8.1) | 9 (10.5) | 12 (13.8) | 10 (11.6) | 0.689 |
| Smoking status, n (%) |  |  |  |  | 0.26 |
| Smoker or ex-smoker | 31 (36.0) | 27 (31.4) | 20 (23.0) | 23 (26.7) |  |
| Non-smoker | 55 (64.0) | 59 (68.6) | 67 (77.0) | 63 (73.3) |  |
| Drinking status, n (%) |  |  |  |  | 0.324 |
| Drinker or ex-drinker | 31 (36.0) | 34 (39.5) | 28 (32.2) | 23 (26.7) |  |
| Non-drinker | 55 (64.0) | 52 (60.5) | 59 (67.8) | 63 (73.3) |  |
| Medicine use before admission, n (%) |  |  |  |  |  |
| Antiplatelet drug | 7 (8.1) | 13 (15.1) | 4 (4.6) | 7 (8.1) | 0.104 |
| Anticoagulant drug | 0 (0) | 1 (1.2) | 4 (4.6) | 2 (2.3) | 0.169 |
| Antihypertensive drug | 32 (37.2) | 38 (44.2) | 45 (51.7) | 40 (46.5) | 0.284 |
| Hypoglycemic drug | 3 (3.5) | 5 (5.8) | 14 (16.1) | 25 (29.1) | <0.001 |
| Statin | 4 (4.7) | 9 (10.5) | 10 (11.5) | 3 (3.5) | 0.109 |
| Time to blood collection (days), median (IQR) | 2 (2,4) | 2 (2, 3) | 2 (2, 4) | 2 (2, 3.3) | 0.605 |
| TC (mmol/L), mean (SD) | 4.6 (1.0) | 4.9 (1.3) | 4.7 (1.1) | 4.9 (1.2) | 0.161 |
| LDL-C (mmol/L), mean (SD) | 2.4 (0.7) | 2.6 (0.9) | 2.4 (0.7) | 2.7 (0.8) | 0.039 |
| eGFR (ml/min/1.73m^2^), median (IQR) | 97.5 (90.1, 106.3) | 99.9 (92.0, 111.1) | 100.0 (92.3, 108.4) | 101.0 (86.6, 113.0) | 0.542 |
| Hematoma volume (ml), median (IQR) | 7.4 (2.9, 15.7) | 7.5 (2.8, 14.4) | 6.4 (3.0, 17.2) | 9.9 (3.2, 21.6) | 0.403 |
| Hematoma location, n (%) |  |  |  |  | 0.638 |
| Lobar | 17(19.8) | 17(19.8) | 16 (18.4) | 18 (20.9) |  |
| Deep | 49 (57.0) | 54 (62.8) | 58 (66.7) | 53 (61.6) |  |
| Infratentorial | 12 (14.0) | 12 (14.0) | 8 (9.2) | 12 (13.9) |  |
| Mixed | 8 (9.3) | 3 (3.5) | 5 (5.7) | 3 (3.5) |  |
| Ventricle extension, n (%) | 15 (17.4) | 16 (18.6) | 31 (35.6) | 40 (46.5) | <0.001 |
| Subarachnoid extension, n (%) | 8 (9.3) | 5 (5.8) | 7 (8.0) | 11 (12.8) | 0.443 |
| Total cSVD burden, median (IQR) | 2 (1,3) | 2 (1,3) | 2 (1,4) | 2 (1, 3.8) | 0.89 |
| Presumed etiology of ICH, n (%) |  |  |  |  | 0.206 |
| HA | 51 (59.3) | 61 (70.9) | 66 (75.9) | 65 (75.6) |  |
| CAA | 12 (14.0) | 10 (11.6) | 10 (11.5) | 8 (9.3) |  |
| Anticoagulation or undetermined cause | 23 (26.7) | 15 (17.4) | 11 (12.6) | 13 (15.1) |  |

HOMA-IR indicates homeostasis model assessment of insulin resistance; Q, quartile; SD, standard deviation; BMI, body mass index; IQR, interquartile range; NIHSS, national institute of health stroke scale; ICH, intracerebral hemorrhage; CI, cerebral infarction; TIA, transient ischemic attack; TC, total cholesterol; LDL-C, low-density lipoprotein-cholesterol; eGFR, estimated glomerular fltration rate; cSVD, cerebral small vessel disease; MRI, magnetic resonance imaging; HA, hypertensive angiopathy; CAA, cerebral amyloid angiopathy.
